# Experimental Study on Bio-Reinforcement of Calcareous Sand through Hydrochloric Acid Solution Precipitation into Cementing Solution

**DOI:** 10.3390/ma16196348

**Published:** 2023-09-22

**Authors:** Zhao Jiang, Renjie Wei, Di Dai, Liangliang Li, Zhiyang Shang, Jiahui Tang, Jie Peng, Ping Li

**Affiliations:** Key Laboratory of Ministry of Education for Geomechanics and Embankment Engineering, Hohai University, Nanjing 210098, China; jiangzhao@hhu.edu.cn (Z.J.); fanyonghua99@163.com (D.D.); lll495107455@163.com (L.L.); shangzy1206@163.com (Z.S.); jhtang5@163.com (J.T.); peng-jie@hhu.edu.cn (J.P.); lipings0110@163.com (P.L.)

**Keywords:** MICP, hydrochloric acid, calcareous sand, UCS, reinforcement efficiency

## Abstract

Microbially induced carbonate precipitation (MICP) technology holds great potential in enhancing soil properties. MICP can be employed to enhance the stability and strength of diverse sandy soil, but it has the shortcoming of low curing efficiency. In response to the identified problem, this study aims to investigate an optimized treatment protocol that involves formulating a cementing solution in a hydrochloric acid (HCl) solution to enhance the solidification rate in the MICP reaction and evaluate its effectiveness. The results indicate that when preparing a 1 M cementing solution in a 0.2 M HCl solution, it promotes the rapid bonding of calcareous sand particles, resulting in an unconfined compressive strength (UCS) of 1312.6 kPa in the sand column after five treatments. Compared to the conventional test group, the experimental group containing HCl exhibited an approximately 1357% increase in UCS. The analysis unveiled the pivotal role of metal ions dissolved from calcareous sand by HCl in enhancing the UCS of MICP-treated calcareous sand. The proposed experimental methodology serves as a valuable tool for designing treatment strategies for MICP-cemented calcareous sand in practical engineering applications.

## 1. Introduction

Soil reinforcement is an important topic in geotechnical engineering. Conventional soil stabilization methods, such as chemical grouting, preloading for consolidation, and grouting stabilization, typically suffer from drawbacks, including prolonged construction periods, high energy consumption, and increased overall expenses. Most chemical materials are also harmful to the environment [1]. Microbially induced carbonate precipitation (MICP), as a promising soil improvement technique, has garnered increasing attention owing to its advantageous features, notably low energy consumption and minimal environmental impact [2,3,4,5,6]. For urea hydrolytic microorganism, it can express the enzyme urease in the process of metabolism, which can facilitate the hydrolysis of urea, then increase the pH value, and finally produce ammonium ions and carbonate ions. Calcium ions are adsorbed onto the negatively charged surface of bacterial cells, when carbonate ions and calcium ions in the solution are supersaturated, CaCO_3_ particles will be formed [7]. The reaction process is shown in Equations (1)–(3). When CaCO_3_ particles precipitate on the surface of sand grains or form interparticle bridges, they inhibit the movement of the sand grains and facilitate their interlocking, thereby achieving the effects of enhancing soil strength, reducing permeability, and providing resistance against external environmental damage.

(1) $$\;CO{\left(NH_{2}\right)}_{2}+2H_{2}O\overset{Urease}{\to }\;2NH_{4}^{+}+CO_{3}^{2-}$$

(2) $$Ca^{2+}+Cell\;\to Cell-Ca^{2+}$$

(3) $$CO_{3}^{2-}+Cell-Ca^{2+}\;\to Cell-CaCO_{3\;}↓$$

According to Mortensen’s research [8], different soil mineralogy can provide favorable nucleation sites for CaCO_3_ precipitation. The rate of CaCO_3_ precipitation can be influenced by soil particle size; it is higher in well-graded and coarser sands compared to finer and poorly graded soils [9]. Consequently, the majority of studies have primarily concentrated on reinforcing sandy soils through MICP. Furthermore, precipitation of CaCO_3_ can provide more ideal nucleation sites within calcium-rich soil particles.

Calcareous sand serves as a prevalent offshore sediment in coastal zones [10,11] and exhibits extensive applications in various domains, such as building foundations, road embankment fill, and aircraft runway construction [12,13]. Nevertheless, calcareous sands exhibit unfavorable properties, such as high porosity and fragility [14,15,16,17]. In response, several scholars have investigated the potential of biomineralization for enhancing the mechanical performance of calcareous sand. For instance, Liu et al. [18] comprehensively investigated various mechanical properties of bio-reinforced calcareous sand. The findings presented herein provide evidence of the feasibility of employing the MICP to enhance the mechanical properties of calcareous sand. Khan et al. [19] explored the potential of MICP for reinforcing calcareous sand, achieving a sand column solidification of up to 20 MPa UCS after 28 days. Deng et al. [20] conducted a study on the factors influencing the properties of MICP-treated calcareous sand. The results indicated optimal pH values within the range of 8–11, the best temperature for urease enzymes exceeding 30 and 35 °C, and the solution concentration significantly affecting the performance of bio-cemented calcareous sand. Numerous studies have investigated the reinforcement of calcareous sand using MICP. However, MICP has its limitations, including a low CaCO_3_ precipitation conversion efficiency and insufficient UCS properties [21,22], particularly when applied to calcareous sand. Compared to quartz sand, the porous structure of calcareous sand poses challenges for achieving efficient strength enhancement via MICP. Furthermore, the limited degree of cementation hinders the practical implementation of MICP for enhancing the strength of calcareous sand [1,23,24].

The existing approach of MICP for reinforcing calcareous sand exhibits shortcomings, such as excessive treatment frequency and low reinforcement efficiency. In order to tackle this issue, Shan et al. [25] proposed the incorporation of activated carbon into the MICP process. The incorporation of activated carbon has shown promising potential in improving both the bacterial retention efficiency and the liquefaction resistance of calcareous sand subjected to MICP. Tang et al. [13] investigated the impact of Mg2+ concentrations on calcareous sand treated with MICP. After soaking in a cementation solution for fourteen days, the samples reached a peak strength of 6.6 MPa. Several studies have sought to improve MICP-treated calcareous sand characteristics by adding fibers [26,27,28,29]. Fang et al. [30] discovered that the addition of fibers resulted in reduced permeability, improved dry density, and increased the UCS of calcareous sand treated with MICP. Despite recent advancements in addressing the limitations of bio-cemented calcareous sand, there is still a need for further exploration into methods to enhance the effectiveness of MICP reinforcement.

This study explores an advanced approach aimed at enhancing the effectiveness of MICP-treated calcareous sand. The method involves preparing the cementing solution with hydrochloric acid (HCl) in MICP tests. To evaluate its effects, sand column specimens were prepared for conducting MICP injection experiments, measuring calcium carbonate content, unconfined compressive strength (UCS), and sample uniformity. Experimental investigations were conducted in aqueous solutions to explore the underlying mechanisms, and techniques, such as scanning electron microscopy (SEM), were employed to observe the formation of CaCO_3_ at the microscale and further elucidate the mechanics.

## 2. Materials

### 2.1. Bacterium

**Figure 1:**
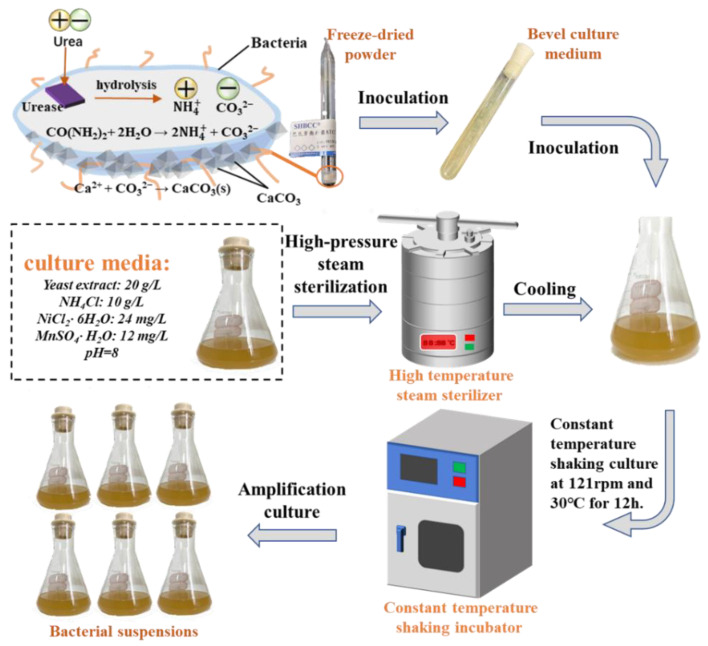
The process of medium preparation and amplification culture of bacteria.

The strain employed in this article was Sporosarcina pasteurii (DSM 33). Sporosarcina pasteurii is a non-pathogenic, Gram-positive aerobic bacterium that was isolated from soil. This bacterium exhibits the capability to induce biomineralization reactions through the production of highly active urease via metabolic processes [31,32]. The process of bacterial culture is illustrated in Figure 1.

### 2.2. Calcareous Sand

**Figure 2:**
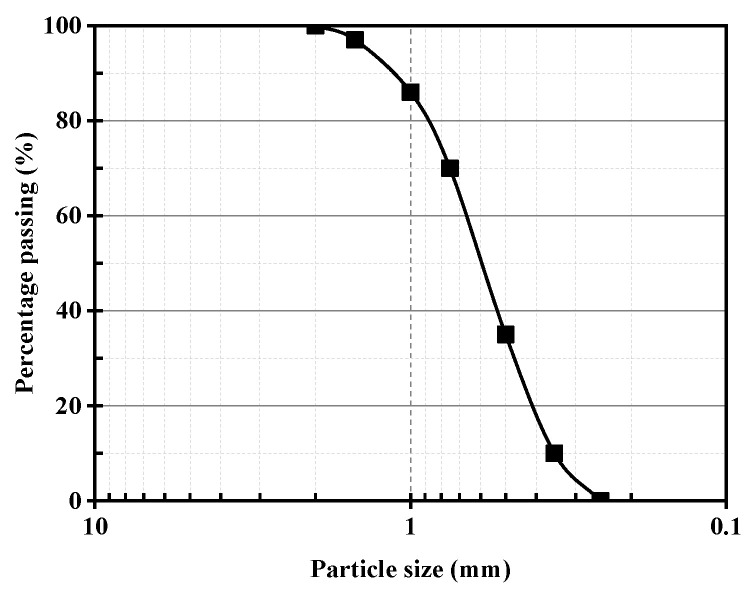
Distribution curve of the calcareous sand used in the experiment.

The calcareous sand utilized in this research was sourced from the South China Sea. The particle size distribution curve is depicted in Figure 2, while Table 1 provides an enumeration of the primary constituents of the calcareous sand.

The test sand had a coefficient of nonuniformity of 2.34, a coefficient of curvature of 0.88, and D50 = 0.65, indicating its classification as poorly graded sand.

### 2.3. Cementing Solution and Hydrochloric Acid

The cementing solution employed in this study comprised chemically pure urea and CaCl_2_. The concentrations of urea and CaCl_2_ were maintained at a consistent level of 1 M. To obtain the mixed solution, the cementing solution was directly prepared in a 0.2 M HCl solution.

## 3. Methods

### 3.1. Sand Specimen Preparation

**Figure 3:**
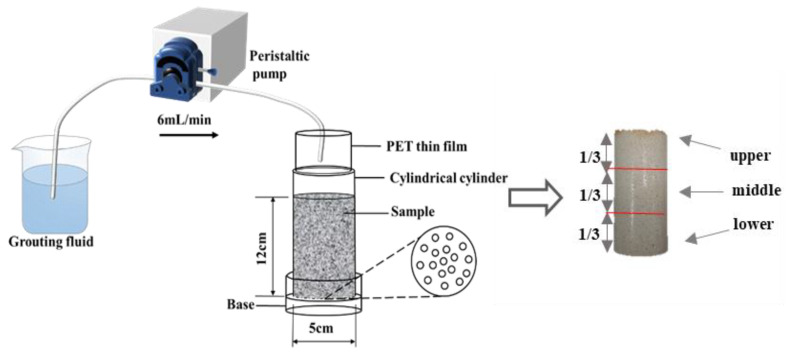
Schematic diagram of the experimental setup.

The calcareous sand employed in the experiment underwent a 12-h rinsing process with deionized water to eliminate surface impurities, followed by oven drying. Subsequently, the sand was introduced into a cylindrical mold, resulting in the formation of a sand column measuring 50 mm in diameter and 120 mm in height [5]. Approximately 318 g of sand was carefully packed and compacted within the mold to attain a controlled dry density of 1.34 g/cm^3^. A visual representation of the experimental setup can be found in Figure 3.

The experimental procedure was as follows: initially, 100 mL of a fixing solution (0.05 M CaCl_2_) was injected to elevate the number of electrolytes in the test sample and promote bacterial adsorption [8]. Next, 100 mL of a bacterial solution was injected, followed by a 6-h adsorption period. Finally, 100 mL of cementing solution was injected, prepared separately using hydrochloric acid and deionized water solutions. The cementing solution was injected every 12 h.

The experimental procedure was conducted with control of the injection speed and temperature to ensure optimal results. Specifically, the injection speed was maintained at a controlled rate of 6 mL/min to prevent inadequate contact time between the sand grains and the bacterial suspension resulting from excessive grouting speed [33]. Moreover, the temperature was held constant at 25 ± 2 °C throughout the experiment to ensure reproducibility and accuracy.

Table 2 presents detailed information about the sand specimens utilized in the study. The control group (Group A_0_) did not contain HCl in the cementing solution. To investigate the impact of HCl addition on the reinforcement of calcareous sand, Group A_1_ was employed, in which the cementing solution was prepared in 0.2 M HCl solution. In Group A_2_, a mixed solution consisting of 1 M CaCl_2_, 1 M urea, and 0.2 M HCl was passed through a calcareous sand specimen without bacteria, and the effluent was collected for use in reinforcing the calcareous sand, following the same procedure as described for the preceding groups.

In all the previously mentioned experiments, the sand columns were extracted after completing the tests and soaked for 24 h to eliminate any biomass and soluble salts. To minimize the potential impact of upper column loss during testing, the sand column dimensions were modified, reducing the base diameter to 5 cm and the height to 10 cm after the injection experiment. Subsequently, the sand column underwent a drying process in order to prepare it for subsequent testing. To ensure the experimental repeatability, three replicates were prepared for each group.

### 3.2. Aqueous Solution Test

In order to examine the reaction mechanism involved in the process of MICP, an aqueous solution test was performed [34,35]. Four experimental groups, labeled S_0_ through S_3_, were established by mixing 100 mL of different testing solutions with 100 mL of bacterial suspension. The purpose of this setup was to examine the impact of varying testing solutions on the bacteria. Group S_2_ had a pH value of 5.68; consequently, the pH of the solution in group S_3_ was adjusted to 5.68 to study the effect of pH on the bacteria. Additionally, four more experimental groups, labeled T_0_ through T_3_, were established to examine the effect of different testing solutions on the MICP reaction. The experimental groups are outlined and detailed in Table 3.

The mixture was stirred using a magnetic stirrer for a duration of 6 h. To ensure the reliability of the results, three replicate tests were conducted for each group.

### 3.3. Test Procedure

#### 3.3.1. Carbonate Precipitation Content

The CaCO_3_ content (*C*) was determined by measuring the weight difference of carbonate precipitation in a dry state before and after MICP reaction [36], as indicated by the following Equation (4):

(4) $$C=\frac{m_{2}-m_{1}}{m_{1}}\times 100\%\;$$

where *m*_1_ represents the initial mass of the specimen prior to reinforcement. Following the MICP reaction, the sand samples were subjected to multiple rinses with deionized water to eliminate any residual solutes. Subsequently, the samples were dried, and their mass was measured as *m*_2_.

#### 3.3.2. UCS

To evaluate the enhancement of physical properties in the bio-cemented calcareous sand samples, a sequence of unconfined compression tests were performed. The UCS tests were conducted using an axial loading rate of 1.0 mm/min, with a loading accuracy of ±0.5% [37].

#### 3.3.3. pH

The pH measurements during the experiments were conducted following the international standard ASTM E70-07(2017) [38].

#### 3.3.4. Bacterial Parameter Test

The biomass concentration was expressed by determining the optical density (*OD*) at a wavelength of 600 nm using a spectrophotometer (*OD*_600_). The actual cell concentration can be quantified utilizing the following equation [39]:

(5) $$Y=8.59\times 10^{7}Z^{1.3627}$$

where Y represents the cell concentration (cell·mL^−1^), and Z corresponds to the measured *OD*_600_ value. It is important to note that the validity of Z is limited to the range of 0.2–0.8. If the measured *OD*_600_ value falls outside this range, appropriate dilution should be performed before conducting the calculations.

Additionally, the bacterial activity in the absence of Ca^2+^ was measured using the conductivity method [40]. We mixed 2 mL of the bacterial suspension to be tested with 18 mL of a 1 M urea solution, and measurements of electrical conductivity were taken at 5-min intervals using a conductivity meter, with three consecutive readings recorded to track changes in conductivity. The urease activity (Mm·urea·hydrolyzed·min^−1^) can be calculated using the following equation:

(6) $$Urease\;activity\left(Mm\cdot urea\cdot ydrolysed\cdot min^{-1}\right)\;=Conductivity\;change\;values\left(mS\cdot min^{-1}\right)\times 11\times Dilution\;times$$

In this study, the initial harvested bacterial suspension had an *OD*_600_ ranging from 1.2 to 1.5, and the urease activity was 7.0 ± 0.5 mM·urea·hydrolyzed·min^−1^.

#### 3.3.5. Microanalysis Test

Scanning electron microscopy (SEM) is a type of electron microscopy that produces high-resolution images of surfaces and the microstructure of materials. The crystal morphologies and mineral compositions of MICP were studied using scanning electron microscopy [5].

## 4. Results

### 4.1. Sand Specimen

Due to the presence of H^+^ in group A_1_, there is actually another reaction that occurs in addition to the MICP, as follows:

(7) $$HCl+Calcareous\;\to \;Soluble\;ions+H_{2}O+CO_{2}\;↑$$

**Figure 4:**
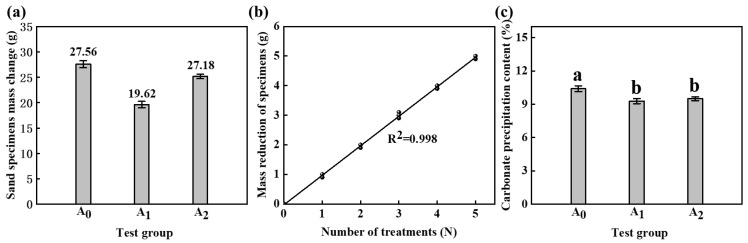
Changes in calcium carbonate and sand specimens. (**a**) Sand specimens’ mass change after 5 MICP treatments. (**b**) Mass reduction in specimens with 0.2 M HCl. (**c**) The estimated value of calcium carbonate production in group A_1_ was compared with other groups. “a” and “b” represent different coefficients of difference.

The reaction of MICP to produce CaCO_3_ increases the mass of the specimen, while the reaction of HCl with calcareous sand decreases the mass of the specimen (Figure 4b). Figure 4a displays the mass change in the sand specimens after five MICP treatments. The estimated value of CaCO_3_ production in group A_1_ can be calculated by adding the value of mass reduction, as demonstrated in Figure 4c.

Upon comparing groups A_0_, A_1_, and A_2_, it can be observed that the presence of HCl lowers the pH, which will reduce the amount of CaCO_3_ produced. Nonetheless, the decrease in CaCO_3_ was relatively low.

**Figure 5:**
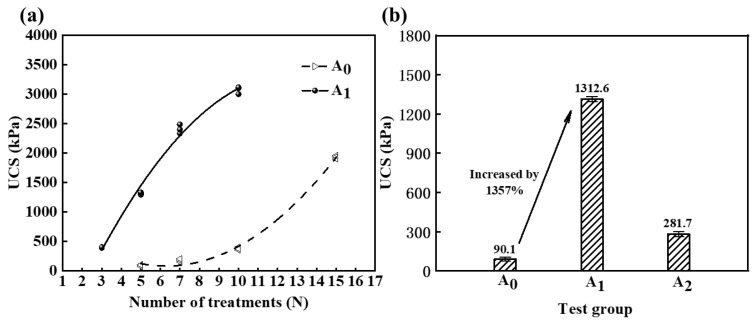
Unconfined compressive strength. (**a**) UCS in groups A_0_ and A_1_ with different number of treatments of cementing solution. (**b**) UCS in groups A_0_, A_1_, and A_2_ with five treatments of cementing solution.

When directly preparing the cementing solution in a HCl solution with a concentration of 0.2 M for the MICP experiment, the generated CaCO_3_ content within the sand column may decrease. However, this approach leads to an increase in the UCS of the MICP-treated calcareous sand. As depicted in Figure 5a, in group A_1_, the calcareous sand particles were bonded together after three treatments of the cementing solution, resulting in a UCS of up to 387 kPa. In contrast, for group A_0_, a significant increase in UCS was observed only after ten treatments, as the accumulation of sufficient CaCO_3_ in the sand specimen enabled bonding. Within the range of 10 treatments with cementing solution, the UCS of the specimen treated with cementing solution prepared in HCl solution after MICP experiments is 8 to 18 times higher than that of the sand column treated with cementing solution prepared in deionized water.

**Figure 6:**
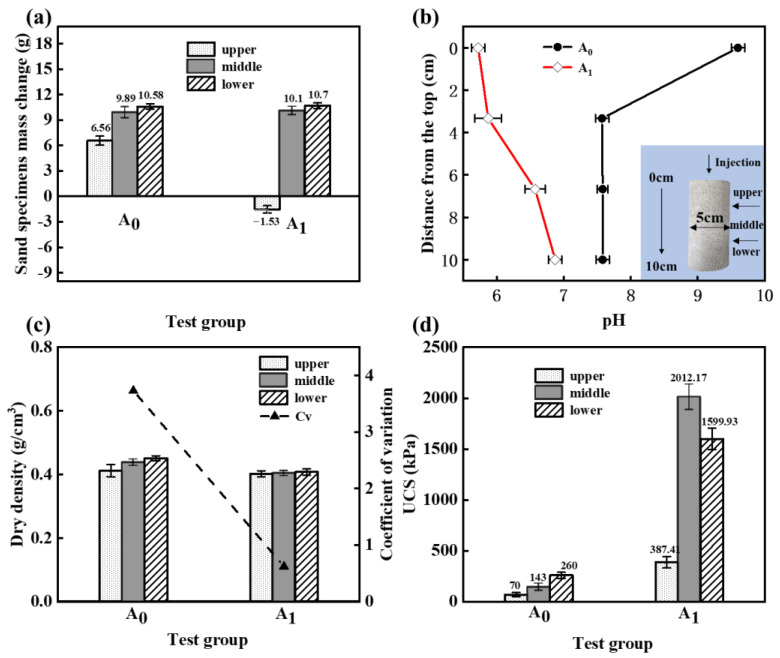
Properties of the upper, middle, and lower parts of sand column. (**a**) Sand specimens’ mass change after 5 MICP treatments for each part. (**b**) pH change during the MICP reaction in different parts. (**c**) Dry density of different parts and coefficient of variation in group A_0_ and group A_1_. (**d**) UCS of different parts in group A_0_ and group A_1_.

In the experiment on MICP for enhancing the strength of calcareous sand, Figure 5b illustrates the results obtained by treating the cementing solution with HCl, with this treatment being applied five times. The UCS of the sand column was found to improve approximately 1357% relative to the conventional test group. However, it is noteworthy that the increase in UCS for group A_2_ was less pronounced than that observed for group A_1_. The mechanism underlying the increase in UCS due to the addition of HCl in the cementing solution is complex and involves both the dissolution of calcareous sand by HCl and the MICP reaction. To investigate this mechanism, the sand column was partitioned into three uniform segments, corresponding to the upper, middle, and lower regions, and the properties of each segment were examined. The results are depicted in Figure 6.

In group A_1_, the mass of the upper part of the sand column decreased by 1.53 g, whereas the mass changes in the middle and lower parts of the sand column were comparable to those of group A_0_. These observations suggest that the reaction between HCl and calcareous sand predominantly took place in the upper part of the sand column, and that the amount of calcareous sand consumed by this reaction was greater than the amount of CaCO_3_ precipitated via the MICP reaction in this region.

In group A_1_, the pH in the sand column increased from top to bottom as the HCl was gradually consumed during the reaction. By contrast, in group A_0_, the pH inside the sand column was relatively stable, except during the initial injection. Overall, the pH in the sand column of group A_1_ was lower than that in group A_0_ (Figure 6b). pH plays a crucial role in regulating bacterial activity, particularly urease activity [41]. To leverage this property, Cheng et al. [35] proposed a new one-phase low-pH injection method that involves reducing the pH of the bacterium solution to inhibit bacterial activity. This enables simultaneous injection of the cementing solution and the bacterium solution, resulting in a more uniform reinforcement of the sand column and improved curing efficacy. To investigate the uniformity of the sand column in this paper, the dry density of each part of the sand column was calculated, and the coefficient of variation (*Cv*) was calculated as shown in the following equation:

(8) $$C_{v}=\frac{S}{Av}\times 100\%$$

where *S* is the standard deviation and *Av* is the mean. A lower value of *Cv* indicates greater uniformity in the reinforcement of the sand column. After removing the unreinforced upper part of group A_1_ resulting from HCl dissolution, Figure 6a,b demonstrate that in the control group, the majority of CaCO_3_ was precipitated at the bottom of the sand column, followed by the middle part, and the least CaCO_3_ was precipitated at the top. This outcome was due to the flushing of bacteria and sediment from the top to the bottom during the injection process. Conversely, in the reinforced part of the sand column in the A_1_ group, CaCO_3_ precipitation was relatively uniform. The greater uniformity can be attributed to lower bacterial activity and the slower MICP reaction resulting from lower pH.

The relationship between CaCO_3_ content and the UCS in the upper, middle, and lower parts of the conventional control group was found to be consistent. In the presence of HCl, the upper part of the sand column exhibited a lower UCS of 387.4 kPa due to HCl dissolution of calcareous sand. Conversely, the middle and bottom parts had a higher UCS, with the middle part having the highest UCS of up to 2012.17 kPa. Interestingly, the test conducted in this paper demonstrated that the relationship between CaCO_3_ and UCS is not strictly linear. The paper achieved a higher UCS despite having lower calcium carbonate content compared to the conventional control group, which is a noteworthy observation. The mechanism behind this phenomenon is further elaborated through the following aqueous solution tests and microscopic experiments.

### 4.2. Aqueous Solution Test

**Figure 7:**
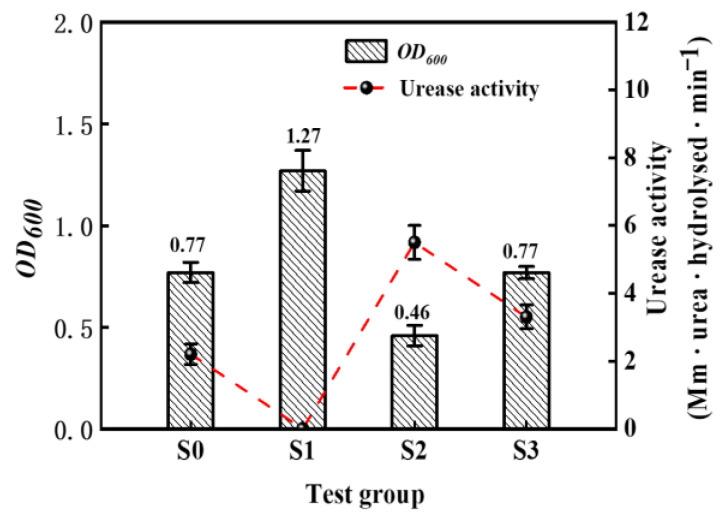
Parameter indicators of bacteria.

The aqueous solution test started by testing the bacterial activity in different solutions, and the results are shown in Figure 7. The *OD*_600_ and activity of the initial bacterial suspension were 1.2 and 7.5 mMurea·hydrolysed·min^−1^, respectively.

After dilution of the bacterial solution with deionized water, the bacteria became less concentrated, leading to a reduction in both the *OD*_600_ and bacterial activity. However, when 0.2 M HCl was added to the bacterial solution, the *OD*_600_ value remained around 1.2, but the bacteria were no longer viable, resulting in a complete loss of activity. When effluent obtained from 0.2 M HCl flowing through a calcareous sand specimen was mixed with the bacterial solution, the *OD_600_* value in the supernatant was found to be 0.46, while the activity was the highest among the four groups at 5.5 mM urea hydrolyzed per minute. Additionally, the *OD*_600_ and activity of group S_3_ were consistent with those of group S_0_, indicating that the pH of the solution (5.63) did not affect the *OD*_600_ or activity of the bacterial suspension.

**Figure 8:**
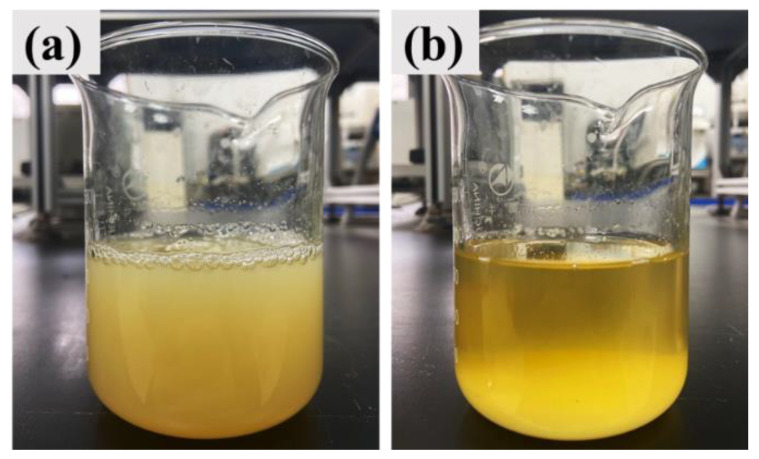
Reaction of group S_2_. (**a**) Magnetic stirring. (**b**) After standing for 6 h.

In group S_2_, the mixture was observed to form flocculated precipitates upon being left to stand for 6 h, as demonstrated in Figure 8. This occurrence signifies the dissolution of metal ions from the calcareous sand subsequent to its reaction with hydrochloric acid, followed by the formation of precipitation in the alkaline environment of the bacterial solution. Notably, the formation of flocculated precipitates serves as a significant influence factor in the improvement of sand column strength.

**Figure 9:**
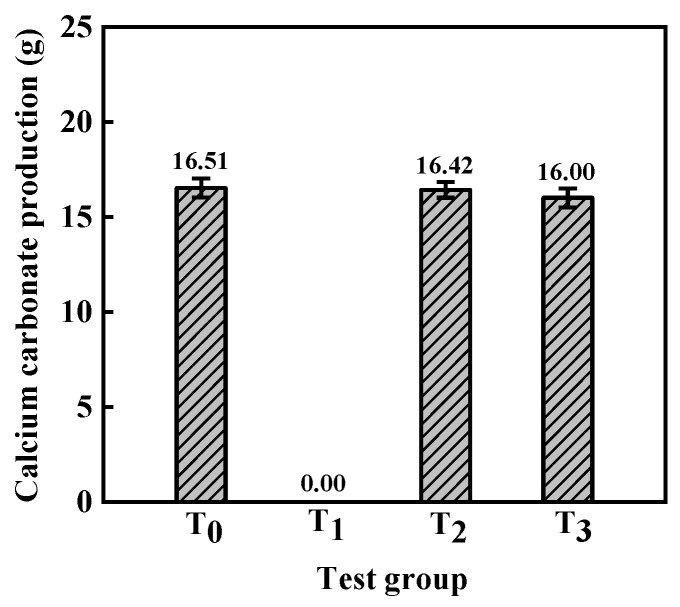
Calcium carbonate production.

Likewise, groups T_0_–T_3_ were established to examine the impact of varying testing solutions on the process of MICP, as depicted in Figure 9. The addition of a cementing solution containing 0.2 M hydrochloric acid to the bacterial solution caused the pH to decrease to a level that inhibited the precipitation of CaCO_3_. Nevertheless, at a pH of 5.63 in the cementing solution, adequate precipitation of CaCO_3_ could be achieved via the MICP reaction. Hence, in the sand column, the pH of the calcium sand was adjusted by reacting it with 0.2 M hydrochloric acid, thereby facilitating the progression of the MICP reaction.

### 4.3. Microanalysis Test

**Figure 10:**
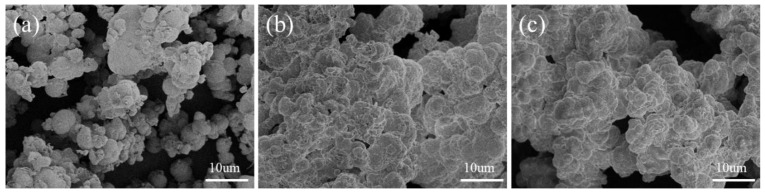
SEM results of aqueous solution products. (**a**) Group T_0_; (**b**) group T_2_; (**c**) group T_3_.

SEM analysis was conducted on the CaCO_3_ obtained from the aqueous solution tests, and the corresponding results are illustrated in Figure 10.

The conventional MICP aqueous solution test yielded calcium carbonate particles that were predominantly spherical in shape, as opposed to the T_2_ and T_3_ groups, which resulted in closely bound particles. Whiffin [40] previously noted that lower pH values led to lower CO_3_^2−^ supersaturation concentrations, resulting in reduced calcium carbonate production rates, larger crystal sizes, and higher interparticle bound strength. These factors ultimately contributed to the strength improvement of the soil. Additionally, the pH value influenced the size and morphology of the CaCO_3_ [42]. The comparison between the groups revealed that the calcium carbonate particles in the T_2_ group were closely bound and had irregular shapes, partly due to the pH reduction.

**Figure 11:**
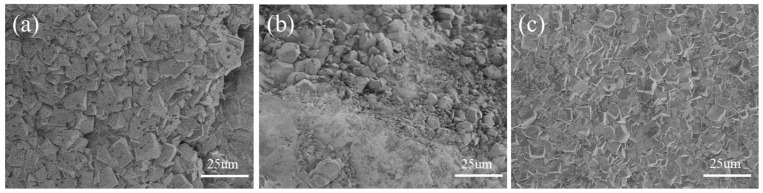
SEM of sand column test. (**a**) Group A_0_; (**b**) group A_2_; (**c**) group A_3_.

**Figure 12:**
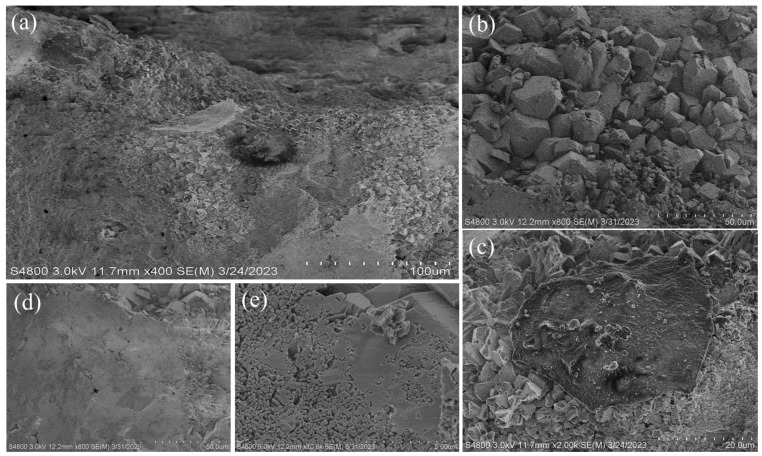
SEM inside sand column of group A_2_. (**a**) Group A_2_, 400×; (**b**) calcium carbonate particles in group A_2_, 800×; (**c**) colloidal material in group A_2_, 2000×; (**d**) calcium carbonate particles and colloidal material in group A_2_, 800×; (**e**) calcium carbonate particles and colloidal material in group A_2_, 10,000×.

It can be observed in Figure 11 that CaCO_3_ in groups A_0_ and A_3_ was cubic and distributed on the surface of sand particles, while in group A_2_, in addition to cubic calcium carbonate, colloidal material was also found. Further details regarding the calcium carbonate production inside the sand specimen in group A_2_ are depicted in Figure 12 for better clarity.

It is obvious that in group A_2_, calcium carbonate particles can be observed intertwined with colloidal material (Figure 12b,d,e). At the same time, the generation of this colloidal material was captured (Figure 12c). Based on previous studies [34], the colloidal substance is aluminum hydroxide gel, and the presence of aluminum hydroxide gel is a key factor in increasing the UCS of the specimen.

When preparing the cementing solution in 0.2 M HCl solution, a reaction occurs between HCl and the calcareous sand, leading to the dissolution of ions from the sand, as illustrated in Table 1. Previous research has demonstrated that the presence of aluminum ions can substantially enhance the reinforcement effect [34]. However, calcareous sand also contains various other ions, such as magnesium ions, iron ions, and strontium ions, whose effects on the MICP reinforced calcareous sand necessitate further investigation.

**Figure 13:**
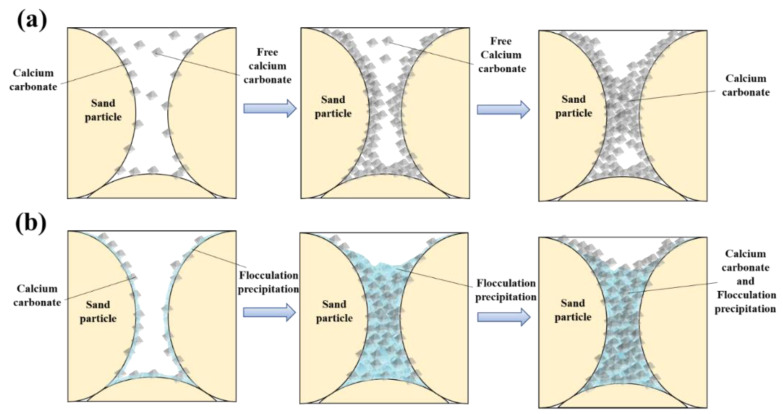
Reaction mechanism. (**a**) Conventional MICP reaction. (**b**) Reaction of hydrochloric acid added to MICP cementing solution.

The dissolved ions flowed through the calcareous sand, and as the HCl was consumed, the dissolved ions existed in the pores of the calcareous sand specimen in the form of flocculated precipitation. Concurrently, the MICP reaction generated calcium carbonate particles, which became adsorbed alongside the flocculated precipitate. The mechanism is shown in Figure 13.

In the case of MICP-treated calcareous sand, the CaCO_3_ predominantly forms aggregates on the particle surfaces (Figure 13a), enveloping the particles, while its presence at the particle-to-particle contacts is limited [23,24]. The porous structure of calcareous sand impedes efficient strength enhancement in MICP-reinforced calcareous sand. In this article, flocculation precipitation changed the CaCO_3_ distribution pattern, allowing calcareous sand particles to bind together more quickly (Figure 13b); macroscopically, the sand column can be reinforced more quickly and with higher strength.

The analysis shows that the significant increase in the UCS of MICP-reinforced calcareous sand was due to the dissolution of metal ion flocs in calcareous sand by HCl. It is noteworthy that Liu et al. [43] used acetic acid to dissolve calcium ions from calcareous sand to extract them for MICP-reinforced calcareous sand and achieved favorable reinforcement results. However, the strength improvement is considerably lower than with the proposed method. In this paper, the UCS of sand column specimens was increased by 1357% after five treatments. Furthermore, in the A_2_ group of this paper, the strength was increased by 68% compared to the control group. There are some similarities between the A_2_ group experiment and Liu et al.’s experiment. It shows that a superior reinforcement effect can be achieved when the HCl dissolution of calcareous sand reaction and MICP reaction are conducted simultaneously, while dissolving calcareous sand followed by MICP reaction cannot effectively introduce metal flocculants into the MICP reaction.

The formulation of a cementing solution in HCl solution for MICP tests, aimed at reinforcing calcareous sand columns, represents a significant advancement in the MICP process, holding substantial implications for engineering applications. This study conducted an initial mechanical investigation into the incorporation of HCl into the MICP test for the reinforcement of calcareous sands. However, further research is needed to assess the influence of other ions in calcareous sands and the effect of low pH on MICP’s effectiveness in reinforcing them.

## 5. Discussion

Currently, microbially induced carbonate precipitation (MICP) is a topic of significant interest in the field of geotechnical engineering. Within this context, enhancing the efficiency of MICP consolidation has emerged as a critical research focus. Numerous researchers have dedicated considerable efforts to this endeavor. Some researchers have altered the distribution of bacteria and the formation of calcium carbonate in soil through various methods. For instance, Shan et al. [25] introduced activated carbon to sand, and Fang et al. [30] incorporated fibers into the sand; both approaches enhance the mechanical characteristics of bio-cemented sand particles. Furthermore, Cheng et al. [42] have enhanced consolidation efficiency by manipulating bacterial activity to achieve a more homogeneous distribution of calcium carbonate within sand grains. Meanwhile, some scholars have introduced additives during the MICP process to enhance the bio-cementation effect through a series of ion reactions [13,34]. This study presents a novel approach, involving the utilization of HCl solution in the preparation of the cementation solution during the MICP process, with the goal of augmenting MICP efficiency by enhancing the cementation solution. This approach results in a substantial improvement in the unconfined compressive strength of MICP-treated sand, accompanied by a reduction in the number of treatment cycles. Importantly, due to variations in target soils, treatment methods, and experimental parameters, direct comparisons between the results of this study and the existing literature pose challenges. The utilization of a HCl solution in the preparation of the cementation solution accomplishes two key objectives: it promotes a more even distribution of calcium carbonate within the enhanced specimen region and precipitates specific metal ions in the sand as flocculants. Consequently, the mechanism by which HCl solution enhances MICP consolidation is exceptionally intricate, and it displays variability in response to alterations in experimental parameters. It is important to note that the findings presented in this study should not be deemed indicative of the ideal consolidation method, and they provide opportunities for further investigation.

## 6. Conclusions

In this study, a novel approach for bio-cemented calcareous sand was explored by formulating a cementing solution in 0.2 M hydrochloric acid solution to improve the MICP reinforcement effect. The effectiveness of this approach was evaluated through an MICP test in an aqueous solution system and calcareous sand reinforcement test, followed by microscopic analysis and comparison using SEM. The key findings are summarized as follows:

(1) Experimental tests were conducted to reinforce calcareous sand using MICP with a cementing solution prepared in a certain concentration of HCl solution. The findings revealed a notable enhancement in the reinforcement efficacy, as evidenced by the UCS reaching 1312.6 kPa following five treatment cycles. Notably, the UCS of the sand column exhibited a remarkable increase of 1357% compared to the control group. The inclusion of hydrochloric acid substantially amplified the reinforcement effect and facilitated the widespread implementation of MICP technology.
(2) The reaction between HCl and calcareous sand leads to insufficient reinforcement in the upper section of the specimen. However, aqueous solution tests demonstrated that MICP can still occur at a pH value of 5.63. The lower pH value within the specimen resulted in a more uniform distribution of CaCO_3_ within the reinforced region of the specimen.
(3) When HCl was involved in the reaction of MICP reinforced calcareous sand, it led to the dissolution of metal ions from the calcareous sand. These metal ions were transformed into flocculated precipitates that changed the distribution pattern of calcium carbonate in the sand column specimens. As a result, the sand particles were able to bond together more quickly, resulting in a rapid reinforcement of the specimen and an increase in UCS.

## Figures and Tables

**Table 1:** Composition of calcareous sand.

**Element**	C&O	Ca	Mg	Al	Si	Sr	F
**Content (%)**	41.827	50.85	2.291	0.695	1.101	0.902	0.581
**Element**	Na	P	S	Fe	Sm	Cl	K
**Content (%)**	0.541	0.383	0.377	0.19	0.165	0.098	0.04

**Table 2:** Grouping table of sand column samples.

Group	Treatment of Cementation Solution	Volume of Bacterial Solution/mL	Volume of Testing Solution per Injection /mL
A_0_	1 M CaCl_2_ + 1 M Urea	100	100
A_1_	1 M CaCl_2_ + 1 M Urea + 0.2 M HCl		
A_2_	The effluent obtained from the mixed solution composed of 1 M CaCl_2_, 1 M urea, and 0.2 M HCl as it flowed through the calcareous sand specimen		

**Table 3:** Details of aqueous solution test.

Group	Testing Solution	Volume of Testing Solution/mL	Volume of Bacterial Solution/mL
S_0_	Deionized water	100	100
S_1_	0.2 M HCl		
S_2_	The effluent obtained from 0.2 M HCl as it flowed through the calcareous sand specimen		
S_3_	Deionized water at pH = 5.63		
T_0_	1 M CaCl_2_ + 1 M urea	100	100
T_1_	1 M CaCl_2_ + 1 M Urea + 0.2 M HCl		
T_2_	The effluent obtained from the mixed solution composed of 1 M CaCl_2_, 1 M urea, and 0.2 M HClas it flowed through the calcareous sand specimen		
T_3_	1 M CaCl_2_ + 1 M urea at pH = 5.63		

## Data Availability

If required, the corresponding author will provide it.
